# Metabolic Changes Induced by High-Fat Meal Evoke Different Microvascular Responses in Accordance with Adiposity Status

**DOI:** 10.1155/2018/5046508

**Published:** 2018-11-01

**Authors:** Priscila Alves Maranhão, Maria das Graças Coelho de Souza, Diogo Guarnieri Panazzolo, José Firmino Nogueira Neto, Eliete Bouskela, Luiz Guilherme Kraemer-Aguiar

**Affiliations:** ^1^Laboratory for Clinical and Experimental Research on Vascular Biology (BioVasc), Biomedical Center, State University of Rio de Janeiro, Rio de Janeiro, RJ 20550-013, Brazil; ^2^Lipids Laboratory (Lablip), Policlínica Piquet Carneiro, State University of Rio de Janeiro, Rio de Janeiro, RJ 20550-003, Brazil; ^3^Obesity Unit, Policlínica Piquet Carneiro, Department of Internal Medicine, Faculty of Medical Sciences, State University of Rio de Janeiro, Rio de Janeiro, RJ 20550-030, Brazil

## Abstract

**Background:**

Frequently, ingestion of lipids exceeds our daily requirements and constantly exposes humans to circulating lipid overload which may lead to endothelial dysfunction (ED), the earliest marker of atherosclerosis. Nailfold videocapillaroscopy (NVC) technique can detect ED on microcirculation. Using NVC, we aimed to demonstrate if metabolic alterations evoked by high-fat meals can act differently on microvascular endothelial reactivity in lean and women with obesity.

**Methods:**

Women, aged between 19 and 40 years, were allocated to control group (CG) and with obesity group (OBG) and were subjected to blood analysis for determination of glucose, total cholesterol (TC), triglycerides (TG), and low-density lipoprotein cholesterol (LDL-c) and high-density lipoprotein cholesterol (HDL-c) and NVC evaluation at fasting and 30, 60, 120, and 180-min after high-fat meal ingestion. NVC technique evaluated microvascular reactivity through the following variables: red blood cell velocity (RBCV) at rest and after 1-min ischemia (maximal red blood cell velocity, RBCV_max_) and time taken to reach it (TRBCV_max_). A* P* value ≤0.05 was considered significant.

**Results:**

High-fat meal promoted a two-phase response in both groups: one until 60-min, associated with glucose and insulin levels, and the other after 120-min, associated with TG levels. Significant differences between groups were observed concerning insulin and HDL-c concentrations only at fasting and TC, TG, and LDL-c levels in all-time points. Regarding microvascular reactivity, RBCV, RBCV_max_, and TRBCV_max_ were significantly different in OBG at 30-min compared to baseline. RBCV_max_ and TRBCV_max_ were significantly different in CG at 30-min and 60-min comparing to fasting. In all-time points, OBG presented RBCV, RBCV_max_ , and TRBCV_max_ significantly different in comparison to CG.

**Conclusion:**

High-fat meal worsened ED on microcirculation in women with obesity and induced impairment of endothelial function in lean ones, reinforcing the association between high-fat meal and atherosclerosis.

## 1. Introduction

Cardiovascular diseases (CVD) are associated with atherosclerosis and are the main cause of death worldwide [1], and their incidence tends to rise as a consequence of world epidemic of obesity [2], which is known to be a risk factor for atherosclerosis [3].

Changes in plasma lipoprotein concentrations are a well-established cause of increased CVD risk [4–6]. However, it does not explain why individuals with normal fasting levels of plasma lipids develop atherosclerosis. Zilversmit [7] was the first to hypothesize that atherogenesis is a postprandial phenomenon since humans spend most part of their days in the postprandial state. Moreover, frequently the ingestion of lipids exceeds the real requirements of the organism, and as a consequence, we are regularly exposed to an overload of circulating lipids [8, 9]. This lipid overload leads to excessive formation of damaging reactive oxygen species (ROS), leukocyte activation, endothelial dysfunction, and foam cell formation [10–13]. The consumption of high-fat meals can turn acute postprandial microvascular endothelial dysfunction into a chronic vascular disease [14].

High-fat diet modifies critical protective functions of endothelium, and as a consequence, the endothelium becomes dysfunctional with a greater tendency for the development of atherosclerosis [15]. The most remarkable feature of endothelial dysfunction is the impairment of endothelium-dependent vasodilatation due to decreased NO bioavailability due to decreased NO synthesis or increased depletion by ROS [16, 17].

Since endothelial dysfunction is considered an early marker of the atherosclerotic process, it is crucial to assess its earliest manifestations in micro- and macrovessels. Recently, we have demonstrated that nailfold videocapillaroscopy (NVC) can detect the first derangements of microvascular function in women with obesity even before the appearance of obesity-related comorbidities [18]. Thus, using this technique, we aimed to demonstrate that metabolic alterations elicited by high-fat meal ingestion can impair microvascular function. We additionally investigated if lean women have different microvascular responses compared to those with obesity.

## 2. Materials and Methods

This is a cross-sectional case-control study approved by the Research Ethical Committee of Hospital Universitário Pedro Ernesto (CAAE: 0190.0.228.000-10). All subjects signed the written informed consent before taking part in the study. This study was registered on Clinical Trials as “Study about the high-fat meal and postprandial lipemia” and numbered as NCT01692327.

### 2.1. Study Design and Participants

Women with obesity were selected from the Outpatient Care Unit for Obesity of the State University of Rio de Janeiro, Brazil. The subjects were divided into two groups: women with obesity (OBG) and controls (CG). We investigated the effects of diet intervention on microvascular reactivity. Before inclusion, participants underwent an assessment visit, which consisted of clinical, biochemical, anthropometric, and body composition evaluations and hemogram. Nineteen women with obesity composed the OBG while 18 lean women were allocated to CG. The main inclusion criteria were women, aged between 19 and 40 years and body mass index (BMI) between 30 and 34.9 kg/m^2^ (OBG) and between 20 and 24.9 kg/m^2^ (CG). Exclusion criteria were as follows: concomitant nutritional or pharmacological interventions; hypothyroidism, metabolic syndrome, any degree of glucose intolerance or diabetes mellitus; arterial hypertension; symptoms or a past history of lactose intolerance; weight gain or reduction by at least 5% of body weight in the six months preceding recruitment; tobacco use, alcohol abuse, and regularly practiced physical activities.

The biochemical analysis comprised the determination of blood levels of insulin, total cholesterol (TC), triglycerides (TG), high-density lipoprotein cholesterol (HDL-c), and thyroid stimulating hormone (TSH) after a 12-hour overnight fast. Fasting and 2-hour after load (75 g, oral) glycemia were also evaluated. Glucose intolerance and metabolic syndrome were defined according to the American Diabetes Association (ADA) criteria [19] and the Joint Interim Statement [20], respectively.

**Figure 1:**
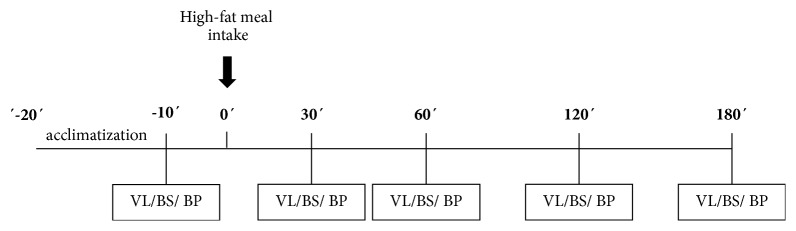
Experimental design. VL: videocapillaroscopy; BS: blood sample collection; BP: blood pressure.

Figure 1 schematizes the periods of the test. On this morning, after a 12 h fast, systolic (SBP) and diastolic (DBP), blood pressures were checked using the standard auscultatory method, and an intravenous catheter was inserted and maintained* in situ* throughout the test for blood samples withdrawal. Time points for blood pressure, microvascular reactivity assessment, and blood samples were as follows: fasting state (baseline) and 30, 60, 120, and 180-min after ingestion of a high-fat meal. Both groups received the same test meal. Participants had been instructed not to ingest fat-rich meals or to practice any physical activity within 24 hours before the test.

### 2.2. Test Meal (High-Fat Meal)

Participants consumed a high-fat meal on the breakfast of the test day, composed of whole milk (200ml), chocolate (10g), margarine (20g),* croissant* (1 unit), cheddar cheese (60g), and salami (31 g). The meal was partially based on Signori and coworkers [21], with some modifications to include more palatable foods [22]. The meal consisted of 691.5 kcal and specifically, 24.8% of carbohydrates, 59.5% of lipids (being 21.9 g of saturated fat), and 15.7% of proteins. The time limit for ingestion of the meal was 10 minutes.

### 2.3. Blood Sample Analysis

Plasma levels of glucose were determined by glucose oxidase colorimetric method. Serum concentrations of TG, TC, and HDL-c were assessed by glycerol phosphate oxidase/peroxidase, cholesterol oxidase/peroxidase, and direct colorimetric methods, respectively. All analysis were performed by commercially available kits appropriated for the Automatic Analyser A25 (BioSystems, Barcelona, Spain), following the protocols provided by the kits manufacturer (BioSystems, Barcelona, Spain). LDL-c was calculated by Friedewald equation [23]. Intra- and interassay coefficient of variation of all analyses described above were below 15% and previously validated [24].

### 2.4. Microvascular Reactivity Assessment

Nailfold videocapillaroscopy (NVC) was performed and analyzed, by the same technician who was blinded to patient's data, according to our standardized, well-validated methodology, described elsewhere in the literature [24]. Images were recorded continuously (final magnification of x680) for later assessment of microvascular variables using Cap Image v7.2 software (Zeintl, Heidelberg, Germany).

Red blood cell velocity (RBCV) at rest, maximal red blood cell velocity (RBCV_max_), and time taken to reach it (TRBCV_max_) were evaluated. RBCV_max_ was measured during post-occlusive reactive hyperemia (PORH) after 1-min ischemia, obtained by a pressure cuff (1 cm wide) placed around the proximal phalanx of the 4th finger and connected to a mercury manometer. We measured basal RBCV three times, and intra-assay coefficient of variation for all measurements ranged from 16.9 to 17.1%. During PORH, each variable was tested once. NVC was repeated on nine subjects on different days, and the obtained inter-assay coefficient of variation ranged from 12.3% to 17.3%.

### 2.5. Statistical Analysis

Statistical analysis was performed by GraphPad® Prism software, version 5. Normal Gaussian distribution was assessed using the Shapiro-Wilk normality test. For Gaussian and non-Gaussian distributions, data were, respectively, expressed by mean±SD and median [1^st^-3^rd^quartiles]. ANOVA repeated measures or Friedman test performed intragroup comparisons. The unpaired* t*-test or the Mann-Whitney* U*-test compared variables between groups. We also proceeded individual regression equations for modeling the relationship between dependent variables (collected at fasting and during postprandial periods) and the independent variable (time after meal intake) which resulted in two variables for each subject that define the linear relationship between the dependent and independent variables, named as slopes and intercepts. The first one represents the steepness of the regression line. The higher the magnitude of the slope, the higher the rate of change. The intercept is the point where this regression line crosses the axis of the dependent variable. After the acquisition of slopes and intercepts, their mean values were used in the analysis. The statistical power for two-tailed comparisons between two independent groups was 0.95 with a* ɑ* error probability of 0.05. TRBCV_max_ (mean±SD) for group 1 and 2 were 11±1.73 and 9±1.35 seconds [25], respectively, estimating a sample size of 17 patients/group and a total sample size of 34 patients. GPower 3.1.10 software was used for power analysis and sample size estimation. A P-value <0.05 was considered significant.

## 3. Results

### 3.1. Clinical, Laboratory, and Body Mass Evaluation

The clinical, laboratory, and body mass characteristics of the study participants are depicted in Table 1. As expected, OBG had significantly greater weight (P<0.001), BMI (P<0.001), waist and hip circumferences (P<0.001), waist-to-hip ratio (WHR, P<0.001), DBP (P<0.01), and fat mass (P<0.001) compared to CG. Additionally, OBG had significantly higher levels of glucose (P<0.05), insulin (P<0.01), TC (P<0.05), LDL-c (P<0.01), and TG (P<0.05) in comparison to CG. On the other hand, OBG had significantly lower muscle mass when compared to CG (P<0.001). No significant differences between groups were found concerning age (P=0.083), height (P=0.73), heart rate (P=0.90), and HDL-c (P=0.33).

### 3.2. Plasma Glucose, Insulinemia, and Lipid Profile before and after the High-Fat Meal Intake

**Figure 2:**
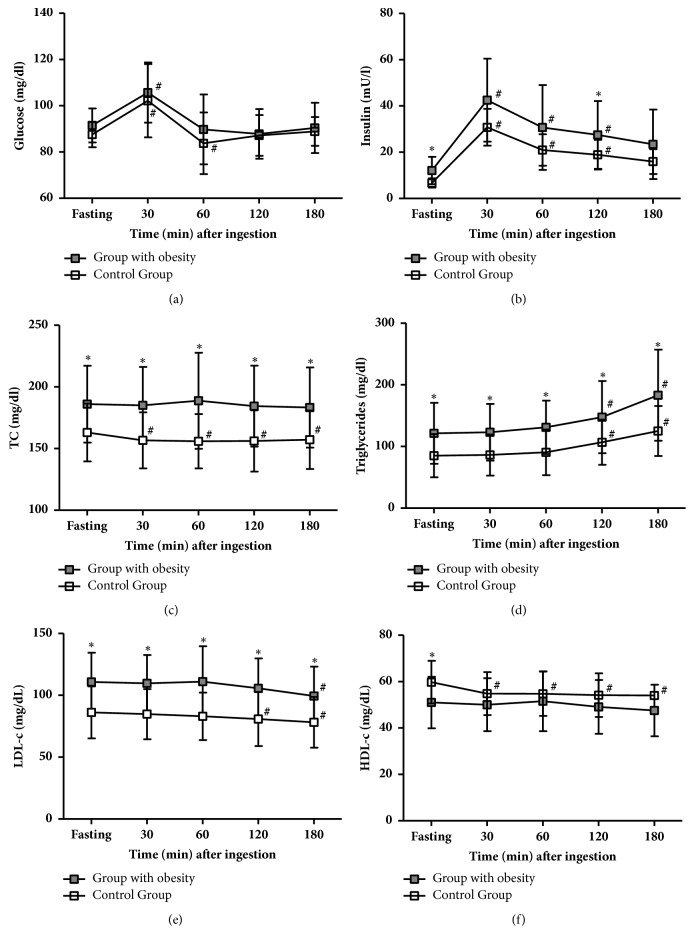
Metabolic responses to a high-fat meal in control group (CG) and with obesity group (OBG). TC: total cholesterol, LDL-c: low-density lipoprotein cholesterol, and HDL-c: high-density lipoprotein cholesterol.

Figure 2 shows the differences between plasma glucose, insulinemia, and lipid profile between groups before and after the high-fat meal intake. In CG, after meal intake, glycemia increased at 30-min followed by a significant reduction at 60-min compared to baseline. Insulinemia also had a peak at 30-min but remained significantly elevated at 60 and 120-min compared to baseline. TC and HDL-c levels decreased significantly after a high-fat meal up to 180-min compared to baseline. LDL-c concentrations remained unchanged until 60-min and decreased significantly afterward when compared to baseline. TG did not change until 60-min, showing a significant progressive increment after that time point compared to baseline. In OBG, insulin and TG levels followed the same trend as in CG. But in contrast, TC and HDL-c concentrations did not decrease after a high-fat meal in OBG. Differently from CG, in the OBG the levels of LDL-c remained unaltered until 120-min and became significantly reduced at 180-min compared to baseline. Additionally, plasma glucose levels increased at 30-min, despite a decrease at 60-min, no statistically significant decrease was observed in OBG.

Considering intergroup analysis, we noticed significant differences between groups in all-time points concerning TC, TG, and LDL-c. Serum levels of insulin in OBG were significantly different from CG at rest and 120-min. In OBG, HDL-c was significantly different from CG at rest. No significant differences between groups were observed regarding plasma glucose levels. No difference in slopes^−1^ (data not shown) was noted for these variables while intercepts for insulin (18.5±4.34 versus 26.96±12.97, P<0.05), TC (159.5±22.66 versus 185.9±30.98, P<0.01), TG (80.70±34.16 versus 120.4±49.93, P<0.01), and LDL-c (86.0±20.78 versus 110.7±23.43 P<0.01) were higher in OBG compared to CG, respectively. No differences in intercept between CG and OBG were found regarding glucose and HDL-c.

In summary, a high-fat meal promoted a comparable two-phase response in both groups: one until 60-min, associated with increases in glucose and insulin, and another one, after 120-min, associated with an increase in triglycerides.

### 3.3. Microvascular Reactivity before and after the High-Fat Meal Intake

Microvascular reactivity differences between groups before and after the high-fat meal intake are shown in Table 2. In all-time points, OBG presented RBCV and RBCV_max_ significantly lower when compared to CG whereas TRBCV_max_ was significantly higher in comparison to CG (P<0.05), pointing to a microvascular dysfunction related to obesity.

RBCV were slower in the OBG at 30-min compared to baseline (P<0.05). RBCV_max_ were significantly slower in CG at 30 min and 60-min and in OBG at 30-min in comparison to baseline (P<0.05). TRBCV_max_ was significantly prolonged in CG at 30-min and 60-min and in OBG at 30-min when compared to baseline (P<0.05).

Although slopes^−1^ for microvascular variables did not add any new finding, intercepts revealed significant differences in microvascular reactivity between groups (P<0.001). In the intergroup comparison, OBG had microvascular dysfunction not only at baseline conditions, expressed by lower RBCV and RBCV_max_ and prolonged TRBCV_max_ compared to CG but also during all postprandial period (intercepts). These observed differences between groups throughout the postprandial period suggest a state of microvascular dysfunction in OBG throughout the tested period compared to CG. Of note, we emphasize that intragroup analysis showed that a high-fat meal elicited different responses between the groups: this diet did not influence RBCV in the CG whereas in OBG it was further reduced at 30-min. Additionally, variables tested during PORH (RBCV_max_ and TRBCV_max_) were influenced by a high-fat meal in both groups. While in CG these responses returned to baseline levels after 60-min, in OBG there was a more significant decrease and an earlier return to baseline levels (after 30-min).

In summary, OBG had microvascular dysfunction that was exacerbated by a high-fat meal while in the CG, an impairment of microvascular function was observed during the first 60-min of ingestion.

## 4. Discussion

To our knowledge this is the first study which demonstrates that high-fat meal elicits an impairment of microvascular function in lean women and aggravates microvascular dysfunction already present in women with obesity at fasting state (baseline conditions).

On this study, we initially demonstrated, as our main finding, that the high-fat meal aggravates microvascular reactivity during the postprandial period in both groups, although in those with obesity it does not recover until the end of the test (180-min). Additionally, during the first 60-min following meal intake we could notice the worst microvascular responses in both groups. Therefore, microvascular dysfunction in the OBG was present during fasting but deteriorated following a high-fat meal, although unexpectedly in lean controls a worse microvascular reactivity occurred after a high-fat meal intake during the first 60 minutes. The OBG participants were age and gender-matched to CG, and according to exclusion criteria they were nondiabetic, nonhypertensive, and they were not diagnosed with metabolic syndrome, suggesting that obesity per se was the primary cause for microvascular dysfunction [26, 27].

Previous studies performed by our group focused on fasting state [18, 24, 26, 27]. Therefore, we decided to investigate if microvascular alterations could be observed during the postprandial period after a high-fat meal since humans are frequently at non fasting state. Furthermore, subjects ingest large amounts of food, generally with high-fat content, consuming between 20 and 70 g of fat by meal [28]. This diet is associated with atherogenic lesions and is also considered an independent cardiovascular disease risk factor [29, 30]. In this study, we have demonstrated that during postprandial state, after a high-fat meal, there is an early functional involvement of the microvascular system, at the capillary level. It is possible to suppose that recurrent damaging bouts to the microvascular system following repeated ingestion of high-fat meals could have further deleterious effects to microcirculation.

During the postprandial state, reduced microvascular reactivity has been observed in obesity after a mixed meal, and this has been explained regarding impaired insulin sensitivity and postprandial hyperglycemia [31, 32]. These results are probably related to visceral adiposity, typically associated with metabolic and vascular complications. However, another study found that microvascular function was not reduced in healthy subjects after a mixed meal, suggesting that the physiological role of the microvascular system (delivery of nutrients and hormones to tissues) during meal ingestion was maintained during the postprandial period [33]. Our data contribute to the understanding of the microhemodynamic behavior of the microcirculation specifically at the capillary level, although we tested it after a high-fat meal. The originality of our data lies on the finding that a high-fat meal affected microvascular reactivity in both groups (controls and women with obesity)

Microvascular reactivity was tested in the fasting state, during the postprandial period, in resting conditions, and during the reactive hyperemia response as well. 1-min ischemia was performed to measure the effect of shear stress during the reactive hyperemic response. At the onset of reperfusion, there is a sharp rise in blood flow followed by a gradual return to its baseline levels, influenced by accumulation of vasodilator metabolites and formation of ROS, customarily washed out, or destroyed by the bloodstream and smooth muscle cell reactivity. During reperfusion, the myogenic response, due to a rapid stretch of microvascular smooth muscle cells, is responsible for the return of blood flow to its baseline values. Microvascular reactivity during reactive hyperemia was associated with glucose metabolism [27] in the fasting state. We have noted that, after a high-fat meal in controls and especially in women with obesity, blood microflow did not return to its baseline values and was further worsened. Hypertriglyceridemia could act negatively on microvascular reactivity inducing a dysfunctional response during the postprandial period, particularly after high-fat meals. However, during our test, triglyceride levels increased only after 120-min while the worst microvascular reactivity was observed during the first 60-min of the test. During this period, insulin and glucose had increased levels among them, and only insulin levels were associated with microvascular reactivity. Therefore, we believe that hyperinsulinemia could better explain these disturbances of microvascular blood flow observed in both groups as a result of meal ingestion during the first hour of the test. Our study cannot exclude the influence of hyperglycemia and also of nonassessed biomarkers (like chylomicrons).

A remarkable point herein observed is the significant declining of microvascular hemodynamics in CG at 30 and 60-min that coincided with a significant reduction of HDL-c serum levels. HDL-c has antioxidant and anti-inflammatory properties, such as decrease of interleukin-1 induced expression of adhesion molecules, reduction of interleukin-8 (the chemotactic factor for neutrophils) expression, and the presence of high content of antioxidant enzymes, such as paraoxonase and glutathione peroxidase, in its structure [34]. Thus, we believe that the decreased HDL-c concentrations contributed to ischemia/reperfusion injury and therefore for the impaired microvascular function at these time points.

Some limitations warrant mention. Ideally, the observation period of the study should be extended to 360-minutes. With this more extended period of observation, the impact of postprandial lipemia would be possibly more apparent not only in blood samples but also on microvascular reactivity.

Our results are restricted to gender since we performed them only in women at menacme. Ketel and coworkers [35] found no differences in microvascular reactivity between phases of the menstrual cycle, although some researchers have suggested this. In this study, we did not perform the tests in the same phase of the menstrual cycle. Only women were recruited for the study because most of the individuals attended at Outpatient Care Unit for Obesity, at our University, were women. Moreover, our experience revealed that women attend frequently the consultations and exams and present greater adherence to clinical trials. In this study, the age range assigned as an inclusion criterion was 19 to 40 years to ensure that women were at menacme and, thus, to avoid menopause and its consequent negative impact on endothelial function due to estrogen deficiency [36], which could be a confounding factor for our analysis.

## 5. Conclusions

A high-fat meal worsened an established impairment of microvascular reactivity in women with obesity but without hypertension, diabetes, and metabolic syndrome and induced it in lean controls. This effect was more pronounced and prolonged in women with obesity, and, in these women, postprandial microvascular dysfunction was associated with higher levels of insulin following high-fat meal ingestion. Our study cannot elucidate the precise mechanism underlying our findings. A possible explanation may be that high-fat meal intake induces hyperinsulinemia, endothelial activation, and inflammation that acutely impair microvascular function in subjects with obesity tested in this study. Our results reinforce the association between high-fat meal intake and atherosclerosis, even in healthy lean subjects, and emphasize the importance of studying microvascular and metabolic changes in the postprandial period.

## Figures and Tables

**Table 1:** Clinical, laboratorial, and body composition characteristics of control group (CG) and obesity group (OBG).

**Variables**	**CG**	**OBG**
Age (y)	27.89 (5.38)	30.74 (4.48)
Weight (kg)	57.82 (6.24)	86.28 (7.84)∗∗∗
Height (m)	1.63 (0.07)	1.63 (0.05)
BMI (kg/m^2^)	21.81 (1.79)	32.31 (1.54)∗∗∗
WC (cm)	79.47 (5.09)	106.4 (9.37)∗∗∗
HC (cm)	100.3 (4.47)	117.5 (6.50)∗∗∗
WHR	0.79 (0.03)	0.91 (0.85)∗∗∗
SBP (mmHg)	109.1 (7.53)	115.8 (13.82)
DBP (mmHg)	69.06 (6.12)	76.74 (9.14)∗∗
Heart rate (bpm)	73.19 (9.56)	72.84 (6.40)
Fat mass (%)	27.88 (3.11)	38.12 (1.78)∗∗∗
Muscle mass (%)	72.14 (3.08)	61.81 (1.75)∗∗∗
Glucose (mg/dl)	81.06 (6.05)	88.79 (13.06)∗
Insulin (mU/l)	8.18 (4.25)	12.74 (4.76)∗∗
TC (mg/dl)	169.40 (32.18)	196.50 (29.24)∗
LDL-c (mg/dl)	86.70 (24.92)	110.40 (27.81)∗∗
TG (mg/dl)	73.72 (37.84)	109.50 (56.42)∗
HDL-c (mg/dl)	67.94 (17.65)	62.37 (16.57)

Data are expressed as mean (SD). BMI: body mass index; WC: waist circumference; HP: hip circumference; WHR: waist-to-hip ratio; SBP: systolic blood pressure; DBP: diastolic blood pressure; TC: total cholesterol; LDL-c: low-density lipoprotein cholesterol; TG: triglycerides; HDL-c: high-density lipoprotein cholesterol. *∗*Significantly different compared to CG group; ∗P<0.05; ∗∗P<0.01; ∗∗∗P<0.001.

**Table 2:** Microvascular reactivity in the control group (CG) and with obesity group (OBG) at fasting and after high-fat meal intake.

Variables	Groups	Fasting	30′	60′	120′	180′	Slope^−1^	Intercept
RBCV (mm/s)	CG	0.326[0.316-0.330]	0.3195[0.3117-0.3291]	0.3237[0.3148-0.3262]	0.3233[0.3113-0.3263]	0.3247[0.3194-0.3305]	2542[-11720, 13700]	0.9697[0.9548, 0.9884]
	OBG	0.3015[0.2866-0.3101]∗	0.2855[0.2723-0.2987]#∗	0.2910[0.2806-0.3053]∗	0.3047[0.2949-0.3195]∗	0.3043[0.2848-0.3167]∗	2043[-93490, 6253]	0.8697[0.8463, 0.8898]∗∗∗
RBCV_max_ (mm/s)	CG	0.3607[0.3483-0.3640]	0.3510[0.3390-0.3577]#	0.3517[0.3390-0.3600]#	0.3583[0.3410-0.3633]	0.3627[0.3573-0.3677]	5604[-14440, 14090]	1.062[1.049, 1.081]
	OBG	0.3300[0.3173-0.3420]∗	0.3200[0.3012-0.3263]#∗	0.3240[0.3107-0.3305]∗	0.3293[0.3178-0.3418]∗	0.3270[0.3197-0.3340]∗	3973[-8382, 22145]	0.9606[0.9475, 0.9824]∗∗∗
TRBCV_max_ (s)	CG	3.0[3.0-4.0]	4.5[4.0-6.25]#	4.0[4.0-5.0]#	4.0[3.0-5.0]	3.5[3.0-4.25]	-96.67[-316.4, 133.8]	3.733[3.267, 4.448]
	OBG	5.0[5.0-7.0]∗	7.0[6.0-8.0]#∗	7.0[5.0-8.25]∗	5.5[5.0-7.0]∗	6.0[5.0-7.0]∗	-61.05[-269.3, 114.9]	6.759[5.798, 7.259]∗∗∗

Data are expressed as median [1st-3rd quartiles]. RBCV: red blood cell velocity; RBCV_max_: red blood cell velocity after 1min ischemia; TRBCV_max_: time taken to reach RBCVmax. ∗Significant difference between groups; ∗P<0.05; ∗∗∗P<0.001. # Significant difference within group.

## Data Availability

The data used to support the findings of this study are available from the corresponding author upon request.
